# Making the Coupled Gaussian Process Dynamical Model Modular and Scalable with Variational Approximations †

**DOI:** 10.3390/e20100724

**Published:** 2018-09-21

**Authors:** Dmytro Velychko, Benjamin Knopp, Dominik Endres

**Affiliations:** Department of Psychology, University of Marburg, Gutenbergstr. 18, 35032 Marburg, Germany

**Keywords:** Gaussian processes, variational methods, movement primitives, modularity

## Abstract

We describe a sparse, variational posterior approximation to the Coupled Gaussian Process Dynamical Model (CGPDM), which is a latent space coupled dynamical model in discrete time. The purpose of the approximation is threefold: first, to reduce training time of the model; second, to enable modular re-use of learned dynamics; and, third, to store these learned dynamics compactly. Our target applications here are human movement primitive (MP) models, where an MP is a reusable spatiotemporal component, or “module” of a human full-body movement. Besides re-usability of learned MPs, compactness is crucial, to allow for the storage of a large library of movements. We first derive the variational approximation, illustrate it on toy data, test its predictions against a range of other MP models and finally compare movements produced by the model against human perceptual expectations. We show that the variational CGPDM outperforms several other MP models on movement trajectory prediction. Furthermore, human observers find its movements nearly indistinguishable from replays of natural movement recordings for a very compact parameterization of the approximation.

## 1. Introduction

Two formidable problems that the human brain has to solve are planning and execution of movements of its body. As a means to simplify these problems while keeping a sufficient degree of control flexibility for a wide range of tasks, modular movement primitives (MP) have been suggested (see [1,2] for reviews). There is no universally accepted definition of the term “movement primitive”. For the purposes of this paper, an MP is a spatiotemporal component of a human (full-body) movement that may be produced by mapping a latent state onto observable variables, such as joint angles. The latent state can be generated by dynamical systems [3] or source functions [4,5]. “Modular” usually refers to the existence of an operation which allows for the spatial, temporal or spatiotemporal combination of (simple) primitives into (complex) movements.

Two prominent examples, where this operation is the linear combination of stereotypical time-courses or muscle-coactivations, are called temporal MP-models [6,7,8,9] or spatial MP-models [10,11]. While these models are inherently modular, the assumption of stereotyped MPs makes it difficult for a control system built out of these primitives to respond to perturbations. A type of MP which can be controlled on-line more easily is the dynamical MP (DMP) [3], which has been developed for robotics applications. In this approach, each primitive is encoded by a canonical second order differential equation with guaranteeable stability properties and learnable parameters. A DMP can generate both discrete (e.g., reaching) and rhythmic (e.g., walking) movements and drives the trajectory of one degree of freedom, e.g., a joint angle. Modularity arises because of the latter property, which might be viewed as an “extreme” form of the modularization that we investigate here, where one movement module might affect several degrees of freedom. Similarly, recent extensions of the DMP framework allow for the reuse of a DMP across end-effectors via kinematical mappings [12] or across tasks [13].

We describe a model that learns MPs composed of coupled dynamical systems and associated kinematics mappings, where both components are learned, thus lifting the DMP’s restriction of canonical dynamics. We build on the Coupled Gaussian Process Dynamical Model (CGPDM) by [14], which combines the advantages of modularity and flexibility in the dynamics, at least theoretically. In a CGPDM, the temporal evolution functions for the latent dynamical systems are drawn out of a Gaussian process (GP) prior [15]. These dynamical systems are then coupled probabilistically, and the result is mapped onto observations by functions drawn from another GP. One drawback of the CGPDM is its fully non-parametric nature, which results in cubic scaling (with the dataset size) of learning complexity and quadratic scaling of MP storage size, i.e., the CGPDM can not be learned from large data sets, and its effective parameterization is not compact. We improve both scalability and compactness with deterministic, sparse variational approximations [16]. In this sparse variational CGPDM, each MP is parameterized by a small set of inducing points (IPs) and associated inducing values (IVs), leading to a compact representation with linear scaling of the training complexity in the number of data points, and constant storage requirements. This compactness is important for real-world applicability of the model, since there might be more primitives than muscles (or actuators) across tasks, as pointed out by Bizzi and Cheung [17]: the motor “code” might be sparse and overcomplete, similar to the sparse codes in early vision [18]. Table 1 provides an overview of the key MP models which we compare in this paper.

Our target application here is human movement modeling, but the vCGPDM could be easily applied to other systems where modularized control is beneficial, e.g., humanoid robotics [9].

We briefly review related work in Section 2 and introduce the vCGPDM in Section 3. The derivation of the variational approximation is outlined in Section 4. In Section 5, we first illustrate the vCGPDM on artificial data. Second, we benchmark the vCGPDM against other MP models. Third, we perform an experiment to quantify the degree of of human-tolerable sparseness in a psychophysics experiment. Fourth, we demonstrate modular movement composition with the vCGPDM. In Section 6, we propose future research directions based on our work.

This paper is a substantially extended version of our earlier conference publication [19].

## 2. Related Work

The Gaussian process (GP) is a machine learning staple for classification and regression tasks [15]. A GP is a prior on functions $R^{Q}\to R$ from a *Q*-dimensional input space to one-dimensional output. By drawing *D* times from the GP, functions from $R^{Q}\to R^{D}$ can be realized. Its advantages include theoretical elegance, tractability and closed-form solutions for posterior densities. Its main disadvantage is cubic runtime scaling with the number of data points. Several solutions have been proposed for this problem. Many of these involve a sparse representation of the posterior process via a small set of IPs, which may [20] or may not be a subset of the data points [21]. If the input space is unobserved, one obtains a GP latent variable model (GPLVM), for which sparse approximations have also been devised [22]. One problem with sparse GP approximations is their tendency to overfit [22], leading to incorrect variance predictions [23]. In that paper, it is also demonstrated that the problem can be alleviated by a variational approximation, which prompted us to develop a similar approach for the CGPDM: as in [24], we extend the sparse GPLVM in time, but we use an autoregressive dynamical system.

**Figure 1:**
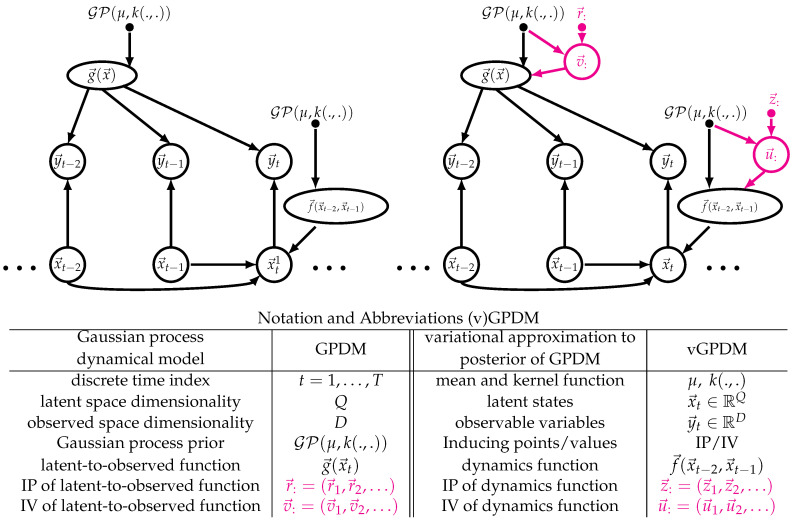
Modular building blocks of the vCGPDM. (**Left**) The Gaussian process dynamical model (GPDM). A latent, second order dynamics model generates a time-series of vector-valued random variables ${\vec{x}}_{t}$ which are drawn from a multivariate Gaussian distribution with mean function $\vec{f}\left({\vec{x}}_{t-2},{\vec{x}}_{t-1}\right)$. The components of this mean function are drawn from a Gaussian Process GP(μ,k(.,.)). Each observable ${\vec{y}}_{t}$ is drawn from multivariate Gaussian distribution with mean function $\vec{g}\left({\vec{x}}_{t}\right)$, which have a Gaussian process prior, too. (**Right**) The GPDM augmented with inducing points and values for a sparse representation of the posterior process [23]. This enables faster variational Bayesian learning and inference, because the augmented GPs are effectively parameterized by these points (here, ${\vec{r}}_{:}$, ${\vec{z}}_{:}$) and corresponding values (here, ${\vec{v}}_{:}$, ${\vec{u}}_{:}$) rather than by the full dataset. They may be thought of as prototypical examples of the corresponding functions, e.g., ${\vec{v}}_{k}=\vec{g}\left({\vec{r}}_{k}\right)$. Slice notation “:” indicates collections of variables. For details, see text.

If the temporal evolution function of this dynamical system is also drawn from a GP, the resulting model is called Gaussian Process Dynamical Model (GPDM), which can be learned by maximum-a-posteriori approximation if the observed dimension *D* is greater than the latent dimension *Q* [25]. Figure 1 (left) shows a graphical model representation of the GPDM, and introduces the related notation which we use throughout the paper. Slices “:” indicate collections of variables along one integer index. Multiple slices refer to collections along multiple indices, e.g., ${\vec{x}}_{:}^{:}$ are the latent variables of all parts and time-steps. The GPDM can model the variability of human movements and has been used for computer animation with style control [26,27,28]. It has also been used with an additional switching prior on the dynamics for motion tracking and recognition [29] and deep variants have been devised [30]. However, with the exception of the coupled GPDM [14,19], all these approaches have a “monolithic” latent space and thus lack the modularity of MPs. One reason for this might be the fact that, for the maximum-a-posteriori approximation to work, the latent space has to be lower-dimensional than the observed space, Q≪D. If, as explained above, we want a modular, possibly overcomplete (i.e., the effective Q>D) set of MPs, we need learning approaches that are robust to overfitting. The works of Frigola et al. [31] indicate that such approaches may be obtained with variational approximations. In the following, we therefore introduce a variational approximation to CGPDM learning and inference based on an approach similar to Frigola et al. [32], but, as in [30], we aim to obviate the need for sampling altogether to allow for fast, repeatable trajectory generation. While deriving a variational approximation is not trivial, we expect it to avoid overfitting and yield a good bound on the marginal likelihood [33]. Figure 1 (right) shows the graphical model of the GPDM augmented by IPs and IVs. This augmentation yields a tractable variational approximation to the GPDM’s posterior [30].

## 3. The Model

The basic building blocks, or “parts”, of the CGPDM are a number of GPDMs run in parallel. In the context of human movement modeling, e.g., they may be thought of as body parts. A part evolves in discrete time t=0,…,T and is endowed with a *Q*-dimensional latent space, a *D*-dimensional observed space and second-order autoregressive dynamics described by a function $\vec{f}\left({\vec{x}}_{t-2},{\vec{x}}_{t-1}\right)$. The component functions ${\left(\vec{f}\left({\vec{x}}_{t-2},{\vec{x}}_{t-1}\right)\right)}_{q}$ have a Gaussian process prior GP(μ,k(.,.)) with mean function μ and kernel k(.,.) Second-order dynamics seem to be a good choice for our target application of human movement modeling [34], but the order can be easily altered simply by concatenating previous states into one larger vector. Let ${\vec{x}}_{t}\in R^{Q}$ the state of latent space of the part at time *t* (see Figure 1, left). This latent state produces observations ${\vec{y}}_{t}\in R^{D}$ via the function $\vec{g}\left({\vec{x}}_{t}\right)$ as well as isotropic Gaussian noise with variance β. The components ${\left(\vec{g}\left({\vec{x}}_{t}\right)\right)}_{d}$ of this function are drawn from a Gaussian process prior, too. GPDMs can be learned from data via a combination of exact marginalization and maximum-a-posteriori learning of the latent dynamics [25].

While the GPDM is a very expressive model, it suffers from poor runtime and memory scaling with the data set size due to its non-parametric nature, which it inherits from the involved GPs. We remedied this problem by an approach pioneered in [23]: augmenting the GPs with inducing points (IPs, here: ${\vec{r}}_{:}$, ${\vec{z}}_{:}$) and associated inducing values (IVs, $\vec{v}$, $\vec{u}$) (see Figure 1, right). These IP/IV pairs might be thought of as prototypical examples of the mappings represented by the corresponding functions, e.g., ${\vec{v}}_{k}^{i}={\vec{g}}^{i}\left({\vec{r}}_{k}^{i}\right)$. Note that the IPs are not drawn from a prior, whereas the IVs are. Hence, the latter are model parameters, whereas the former are not: IPs are merely parameters of the approximation, or “variational parameters”. This augmentation allows for the derivation of a closed-form evidence lower bound (ELBO) on the marginal likelihood of the model.

**Figure 2:**
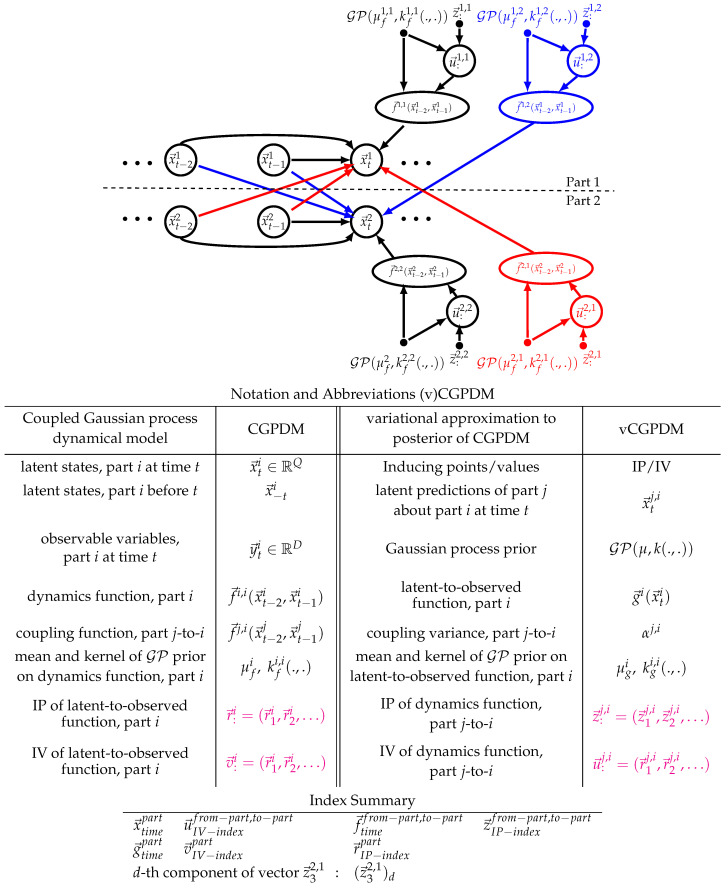
(**Top**): Graphical model representation of the augmented Coupled Gaussian Process Dynamical Model (vCGPDM). Shown is a model with two parts, indicated by the superscripts i,j∈{1,2}. Each part is a vGPDM (see Figure 1), augmented with inducing points ${\vec{z}}_{:}^{i,j}$ and values ${\vec{u}}_{:}^{i,j}$ for variational inference and learning, and modular re-composition of learned GPDM components. Observed variables ${\vec{y}}_{t}^{i}$ and latent-to-observed mappings ${\vec{g}}^{i}\left({\vec{x}}_{t}^{i}\right)$ omitted for clarity. The vGPDMs interact by making predictions about each other’s latent space evolution via functions ${\vec{f}}^{i,j}\left({\vec{x}}_{t-2}^{i},{\vec{x}}_{t-1}^{i}\right)$, here ${\vec{f}}^{1,2}\left(\right)$ and ${\vec{f}}^{2,1}\left(\right)$. Their predictions are product-of-experts combined with the predictions made by each GPDM’s dynamics model (functions ${\vec{f}}^{i,i}\left({\vec{x}}_{t-2}^{i},{\vec{x}}_{t-1}^{i}\right)$). (**Bottom**): Notation and index summaries.

In a CGPDM, the latent spaces of the parts are coupled to each other. We index parts by superscripts i=1,…,M. The index notation in such models can be confusing for first-time readers, we provide a notation and index overview in the tables below Figure 2. In our target application, the coupling may reflect the influences which parts of an articulated body have on each other during the execution of a movement. The coupling is implemented by having the parts make Gaussian-distributed predictions ${\vec{x}}_{t}^{i,j}$ about each other’s latent states with means generated by M×M many mean coupling functions ${\vec{f}}^{i,j}\left({\vec{x}}_{t-2}^{i},{\vec{x}}_{t-1}^{i}\right)$ and coupling variances $\alpha^{i,j}$. *i* indexes the origin part of a coupling, and *j* its target. Thus, ${\vec{f}}^{i,i}$ refers to the dynamics function for part *i*. The components of ${\vec{f}}^{i,j}\left({\vec{x}}_{t-2}^{i},{\vec{x}}_{t-1}^{i}\right)$ are drawn from GPs. As described in [14], these predictions are combined with a product-of-experts construction (PoE [35]), including the predictions which a part makes about its own future. A product-of-experts construction forces the experts to agree on one prediction (Equation (1), left), which amounts to multiplying the individual predictions (Equation (1), right) and renormalizing (Equation (1), middle):

(1) $$p\left({\vec{x}}_{t}^{j}|{\vec{f}}^{:,j}\left({\vec{x}}_{t-2}^{i},{\vec{x}}_{t-1}^{j}\right),\alpha^{:,j}\right)\;=\;\frac{\exp\left[-\frac{1}{2\alpha^{j}}{\left({\vec{x}}_{t}^{j}-\alpha^{j}\sum_{i}\frac{{\vec{f}}^{i,j}\left({\vec{x}}_{t-2}^{i},{\vec{x}}_{t-1}^{j}\right)}{\alpha^{i,j}}\right)}^{2}\right]}{{\left(2\pi \alpha^{j}\right)}^{\frac{Q^{j}}{2}}}\propto \prod_{i}N\left({\vec{x}}_{t}^{j}|{\vec{f}}^{i,j}\left({\vec{x}}_{t-2}^{i},{\vec{x}}_{t-1}^{i}\right),\alpha^{i,j}\right)$$

where $\alpha^{j}={\left(\sum_{i}{\left(\alpha^{i,j}\right)}^{-1}\right)}^{-1}$.

To understand the function of the $\alpha^{i,j}$, consider the form of Equation (1): the smaller a given variance, the more important the prediction of the generating part. We optimize the $\alpha^{i,j}$ during learning, letting the model discover which couplings are important for predicting the data. In other words, whenever an $\alpha^{i,j}$ is small compared to $\alpha^{i^{\prime }\neq i,j}$, then part *i* is able to make a prediction about part *j* with (relatively) high certainty. Furthermore, the $\alpha^{i,j}$ can be modulated after learning to generate new movements, as shown below.

In the following, we denote all relevant timesteps before time *t* with subscript −t, e.g., ${\vec{x}}_{-t}^{j}=\left({\vec{x}}_{t-2}^{j},{\vec{x}}_{t-1}^{j}\right)$ for a second-order dynamics model. We showed in [14] that the individual predictions of part *i* about part *j*, ${\vec{x}}_{t}^{i,j}$ can be exactly marginalized, leading to a GPDM-like model for each part with a dynamics kernel given by the $\alpha_{i,j}$-weighted mean of the individual coupling kernels:

(2) $$k_{f}^{j}\left({\vec{x}}_{-t}^{1},{\vec{x}}_{-t}^{1\;\prime },\ldots ,{\vec{x}}_{-t}^{M},{\vec{x}}_{-t}^{M\;\prime }\right)={\alpha^{j}}^{2}\sum_{i=1}^{M}\frac{k_{f}^{i,j}\left({\vec{x}}_{-t}^{i},{\vec{x}}_{-t}^{i\;\prime },{\vec{x}}_{-t}^{j},{\vec{x}}_{-t}^{j\;\prime }\right)}{{\alpha^{i,j}}^{2}}$$

However, doing so results in a model which lacks modularity: after learning, it is difficult to separate the parts from each other, and recombine them for the production of new movements that were not in the training data. We facilitate this modular recombination by restating CGPDM learning such that we can keep an explicit, sparse representation of the coupling functions. Another reason for a sparse representation is that the CGPDM exhibits cubic run time scaling with the data points, which it inherits from the composing GPDMs. To remedy these problems, we follow the treatment in [16,30]: we augment the model with IPs ${\vec{r}}_{:}^{i}$ and associated IVs ${\vec{v}}_{:}^{i}$ such that $g^{i}\left({\vec{r}}_{k}^{i}\right)={\vec{v}}_{k}^{i}$ for the latent-to-observed mappings $g^{i}\left(\right)$. Then, we condition the probability density of the function values of $g^{i}\left(\right)$ on these IPs/IVs, which we assume to be a sufficient statistic. Likewise, we reduce the computational effort for learning the dynamics and coupling mappings by inducing them through ${\vec{z}}_{:}^{i,j}$ and ${\vec{u}}_{:}^{i,j}$ (also known as “dynamics IPs/IVs”). See Figure 2 for a graphical representation of the augmented model.

Besides introducing IPs, computing an ELBO requires a simplifying assumption about the latent state posterior, which is intractable. We choose a posterior distribution *q* over the latent states ${\vec{x}}_{t}^{i}$ that factorizes across time steps 0,…,T, parts 1,…,M and latent dimensions $1,\ldots ,Q^{i}$ within parts. Furthermore, we assume that the individual distributions are Gaussian:

(3) $$q\left({\vec{x}}_{0}^{1},\ldots ,{\vec{x}}_{T}^{M}\right)=\prod_{t=0}^{T}\prod_{i=1}^{M}\prod_{q=1}^{Q^{i}}q\left({\left({\vec{x}}_{t}^{i}\right)}_{q}\right)\;;\;\;q\left({\left({\vec{x}}_{t}^{i}\right)}_{q}\right)=N\left(\mu_{t,q}^{i},\sigma_{t,q}^{2,i}\right).$$

While this approximation is clearly a gross simplification of the correct latent state posterior, with the right choice of kernels, an ELBO can now be computed. Our approximation assumption (Equation (3)) seems appropriate for human movement data, see Section 5. Whether it is also useful for other data remains to be determined.

As for a tractable kernel, we decided to use an ARD (automatic relevance determination) squared exponential kernel [36] for every part-*i*-to-*j* prediction GP:

(4) $$k^{i,j}\left({\vec{x}}_{-t}^{i},{\vec{x}}_{-t}^{i\;\prime }\right)=\exp\left(-\frac{1}{2}\sum_{t\in -t}\sum_{q}^{Q^{i}}\frac{{\left({\left({\vec{x}}_{t}^{i}\right)}_{q}-{\left({\vec{x}}_{t}^{i}{}^{\prime }\right)}_{q}\right)}^{2}}{\lambda_{q}^{i,j,t}}\right).$$

and a radial basis function kernel for the latent-to-observed mappings. Next, we outline the key steps of the derivation of the ELBO.

## 4. Computing an Evidence Lower Bound for the vCGPDM: An Overview

In this section, we provide an overview of the derivation of the evidence lower bound (ELBO) for the vCGPDM; for details, the reader is referred to Appendix C. We construct a sparse variational approximation by augmenting each of the M×M dynamics and coupling mappings ${\vec{f}}^{i,j}\left(\right)$ with IPs and IVs. The variational distribution of the latent variables, $q\left({\vec{x}}_{:}^{:}\right)=q\left({\vec{x}}_{1}^{1},\ldots ,{\vec{x}}_{T}^{M}\right)$ factorizes according to Equation (3). We let $q({\vec{u}}_{:}^{i})$ and $q({\vec{v}}_{:}^{i})$ be unconstrained distributions, which will turn out to be multivariate Gaussians. In the following, we denote the coupling function values at *t* with ${\vec{f}}_{t}^{i,j}=f^{i,j}\left({\vec{x}}_{-t}^{i}\right)$ and likewise ${\vec{g}}_{t}^{i}=g^{i}\left({\vec{x}}_{t}^{i}\right)$. The factor structure of the joint density of the augmented model follows from the graphical model (see Figure 1 and Figure 2):

(5) $$p\left({\vec{y}}_{:}^{:},{\vec{g}}_{:}^{:},{\vec{v}}_{:}^{:},{\vec{x}}_{:}^{:},{\vec{f}}_{:}^{:,:},{\vec{u}}_{:}^{:,:}|{\vec{z}}_{:}^{:,:},{\vec{r}}_{:}^{:}\right)=p\left({\vec{y}}_{:}^{:}|{\vec{g}}_{:}^{:}\right)p\left({\vec{g}}_{:}^{:}|{\vec{x}}_{:}^{:},{\vec{v}}_{:},{\vec{r}}_{:}^{:}\right)p\left({\vec{v}}_{:}|{\vec{r}}_{:}^{:}\right)p\left({\vec{x}}_{:}^{:},{\vec{f}}_{:}^{:,:}|{\vec{u}}_{:}^{:,:},{\vec{z}}_{:}^{:,:}\right)p\left({\vec{u}}_{:}^{:,:}|{\vec{z}}_{:}^{:,:}\right)$$

Note that we marginalized (most of) the functions $f^{:,:}\left(\right)$ here, keeping only their values at the latent points ${\vec{x}}_{:}^{:}$ and at the IPs. Hence the dependence of ${\vec{g}}_{:}^{:}$ on ${\vec{r}}_{:}^{:}$. Likewise, ${\vec{f}}_{:}^{:,:}$ depends on ${\vec{z}}_{:}^{:,:}$. For easier notation, we omit spelling out the dependence of the IVs on the IPs in the following. Thus,

(6) $$\begin{array}{cc} p({\vec{y}}_{:}^{:}|{\vec{g}}_{:}^{:}) & =\prod_{i=1}^{M}\prod_{d=1}^{D_{i}}p\left({\left({\vec{y}}_{:}^{i}\right)}_{d}|{\left({\vec{g}}_{:}^{i}\right)}_{d}\right) \end{array}$$

(7) $$\begin{array}{cc} p({\vec{g}}_{:}^{:}|{\vec{x}}_{:}^{:},{\vec{v}}_{:}^{:}) & =\prod_{i=1}^{M}\prod_{d=1}^{D_{i}}p\left({\left({\vec{g}}_{:}^{i}\right)}_{d}|{\vec{x}}_{:}^{i},{\left({\vec{v}}_{:}^{i}\right)}_{d}\right) \end{array}$$

(8) $$\begin{array}{cc} p\left({\vec{v}}_{:}^{:}\right)=\prod_{i=1}^{M}p\left({\vec{v}}_{:}^{i}\right) & ;\;\;p\left({\vec{u}}_{:}^{:,:}\right)=\prod_{i=1}^{M}\prod_{j=1}^{M}p\left({\vec{u}}_{:}^{j,i}\right). \end{array}$$

where Equation (6) follows from the assumption of independent observation noise. Equation (7) is a consequence of the Gaussian process prior on the $g^{i}\left(\right)$, which makes the components of ${\vec{g}}_{t}^{i}$ independent. The density of the latent variables and the individual parts’ predictions can be factorized as:

(9) $$p\left({\vec{x}}_{:}^{:},{\vec{f}}_{:}^{:,:}|{\vec{u}}_{:}^{:,:}\right)=p\left({\vec{x}}_{:}^{:}|{\vec{f}}^{:,:},{\vec{u}}_{:}^{:,:}\right)p\left({\vec{f}}_{:}^{:,:}|{\vec{u}}_{:}^{:,:}\right)$$

with

(10) $$\begin{array}{cc} p({\vec{x}}_{:}^{:}|{\vec{f}}_{:}^{:,:},{\vec{u}}_{:}^{:,:}) & =\prod_{t=2}^{T}\prod_{i=1}^{M}p\left({\vec{x}}_{t}^{i}|{\vec{f}}_{t}^{:,i},\alpha^{:,i}\right) \end{array}$$

(11) $$\begin{array}{cc} p({\vec{f}}_{:}^{:,:}|{\vec{u}}_{:}^{:,:}) & =\prod_{t=2}^{T}\prod_{i=1}^{M}\prod_{j=1}^{M}p\left({\vec{f}}_{t}^{j,i}|{\vec{f}}_{1:t-1}^{j,i},{\vec{x}}_{0:t-1}^{j},{\vec{u}}_{:}^{j,i}\right) \end{array}$$

where Equation (10) follows from the graphical model and the product-of-experts construction (Equation (1)). An empty slice (t<2 for a second-order dynamics model) implies no conditioning. The first two latent states at t=0,1 are drawn from independent Gaussians, $\prod_{i=1}^{M}p\left({\vec{x}}_{0}^{i}\right)p\left({\vec{x}}_{1}^{i}\right)$. Equation (11) is one possible way of factorizing the augmented Gaussian process prior on the coupling function values: when ${\vec{f}}^{i,j}\left(\right)$ is marginalized, the function values at time *t* depend on all past function values and latent states. We use this particular factorization for analytical convenience. Note that the dependence of the right hand side of Equation (11) on ${\vec{x}}_{0:t-1}^{j}$ does not contradict the factorization order of Equation (9), because it depends only on latent variables from timesteps prior to *t*. Furthermore, we choose the following proposal variational posterior:

(12) $$q\left({\vec{g}}_{:}^{:},{\vec{x}}_{:}^{:},{\vec{v}}_{:}^{:},,{\vec{f}}_{:}^{:,:},{\vec{u}}_{:}^{:,:}\right)=p\left({\vec{g}}_{:}^{:}|{\vec{x}}_{:}^{:},{\vec{v}}_{:}^{:}\right)q\left({\vec{v}}_{:}^{:}\right)p\left({\vec{f}}_{:}^{:,:}|{\vec{u}}_{:}^{:,:}\right)q\left({\vec{x}}_{:}^{:}\right)q\left({\vec{u}}_{:}^{:}\right)$$

with $p({\vec{g}}_{:}^{:}|{\vec{x}}_{:}^{:},{\vec{v}}_{:}^{:})$ given by Equation (7), $p({\vec{f}}_{:}^{:,:}|{\vec{u}}_{:}^{:,:})$ by Equation (11) and $q({\vec{x}}_{:}^{:})$ by Equation (3). The densities $q(\vec{v})$ and $q(\vec{u})$ are unconstrained except for normalization. With these distributions, we derive the standard free-energy ELBO [36], denoting $\Theta =({\vec{x}}_{:}^{:},{\vec{u}}_{:}^{:,:},{\vec{f}}_{:}^{:,:},{\vec{v}}_{:}^{:},{\vec{g}}_{:}^{:})$:

(13) $$\log p\left({\vec{y}}_{:}^{:}\right)\geq L\left(\Theta \right)=\int d\Theta q\left(\Theta \right)\log\left(\frac{p({\vec{y}}_{:}^{:},\Theta )}{q(\Theta )}\right)$$

exploiting the assumption that the IPs ${\vec{r}}_{:}^{i}$ and IVs ${\vec{v}}_{:}^{i}$ are sufficient statistics for the function values ${\vec{g}}_{:}^{i}$. As we explain in detail in Appendix C, after canceling common factors in the variational posterior (Equation (12)) and the joint model density (Equation (5), cf. [16]), we find that the ELBO can be decomposed into one summand per part that describes the quality of the kinematics mapping (latent-to-observed) $L_{kin}^{i}$, and one summand for the dynamics $L_{dyn}$:

(14) $$L\left(\Theta \right)=\sum_{i=1}^{M}L_{kin}^{i}+L_{dyn}$$

where

(15) $$L_{kin}^{i}=\sum_{d=1}^{D}\int d{\vec{x}}_{:}^{i}\;d{\left({\vec{v}}_{:}^{i}\right)}_{d}\;d{\left({\vec{g}}_{:}^{i}\right)}_{d}\;p\left({\left({\vec{g}}_{:}^{i}\right)}_{d}|{\vec{x}}_{:}^{i},{\left({\vec{v}}_{:}^{i}\right)}_{d}\right)q\left({\vec{x}}_{:}^{i}\right)q\left({\left({\vec{v}}_{:}^{i}\right)}_{d}\right)\log\frac{p({\left({\vec{y}}_{:}^{i}\right)}_{d}|{\left({\vec{g}}_{:}^{i}\right)}_{d})}{q({\left({\vec{v}}_{:}^{i}\right)}_{d})}.$$

is—up to the Shannon entropy of approximating posterior of the latent dynamics variables $H\left(q\left({\vec{x}}_{:}^{i}\right)\right)=-\int d{\vec{x}}_{:}^{i}q\left({\vec{x}}_{:}^{i}\right)\log\left(q({\vec{x}}_{:}^{i})\right)$—equal to the Bayesian GPLVM ELBO of [16]. The remaining integral

(16) $$\begin{array}{cc} L_{dyn} & =\int d{\vec{u}}_{:}^{:,:}\;q\left({\vec{u}}_{:}^{:,:}\right)\left[\sum_{t=2}^{T}\int d{\vec{x}}_{1:t}^{:}\;q\left({\vec{x}}_{1:t}^{:}\right)\left(\int d{\vec{f}}_{t}^{:,:}\;p\left({\vec{f}}_{t}^{:,:}|{\vec{f}}_{2:t-1}^{:,:},{\vec{x}}_{0:t-1}^{:},{\vec{u}}_{:}^{:,:}\right)\log p\left({\vec{x}}_{t}^{:}|{\vec{f}}_{t}^{:,:},\alpha^{:,:}\right)\right)\right]\\ & +\int d{\vec{u}}_{:}^{:,:}q\left({\vec{u}}_{:}^{:,:}\right)\log\frac{p({\vec{u}}_{:}^{:,:})}{q({\vec{u}}_{:}^{:,:})}+\int d{\vec{x}}_{0:1}^{:}q\left({\vec{x}}_{0:1}^{:}\right)\log p\left({\vec{x}}_{0:1}^{:}\right)+H\left(q\left({\vec{x}}_{:}^{:}\right)\right) \end{array}$$

is derived in detail in Appendix C. Briefly, we use the assumption that the IPs and IVs ${\vec{z}}_{:}^{j,i}$ and ${\vec{u}}_{:}^{j,i}$ are sufficient statistics for the function values ${\vec{f}}_{t}^{j,i}$. Optimizing with respect to $q({\vec{u}}^{:,:}:$) can be carried out in closed form using variational calculus and yields

(17) $$L_{dyn}\left(\Theta \right)\geq \log\int p\left({\vec{u}}_{:}^{:,:}\right)\exp\left(C\left({\vec{u}}_{:}^{:,:}\right)\right)d{\vec{u}}_{:}^{:,:}+H\left(q\left({\vec{x}}_{:}^{:}\right)\right)$$

where $C({\vec{u}}_{:}^{:,:})$ is given by Equation (A31). The inequality is due to the sufficient statistics assumption, which introduces another approximation step that lower-bounds $L_{dyn}$. We now have all the ingredients to compute the ELBO for the whole model, and learn it.

## 5. Results

We used the machine-learning framework Theano [37] for automatic differentiation in Python 2.7 (Python Software Foundation. Python Language Reference, version 2.7. Available at http://www.python.org) to implement the model, and learned via optimization of the ELBO with the scipy.optimize.fmin_l_bfgs_b routine [38]. Latent space trajectories were initialized with PCA. We obtained the best ELBOs by first optimizing all parameters jointly, followed by a blocked optimization procedure. We optimize three groups of parameters: latent points and variances, kernel parameters and couplings, and IPs. The number of iterations of the blocked procedure depended on the application; we provide details in the sections below.

The advantage of the sparse approximations in the vCGPDM is that memory consumption of the model is greatly reduced. However, this approximation might also introduce errors, along with the fully factorized latent posterior (Equation (3)). We tried to quantify these errors in a cross-validatory model comparison, and in a human perception experiment.

### 5.1. Synthetic Data

**Figure 3:**
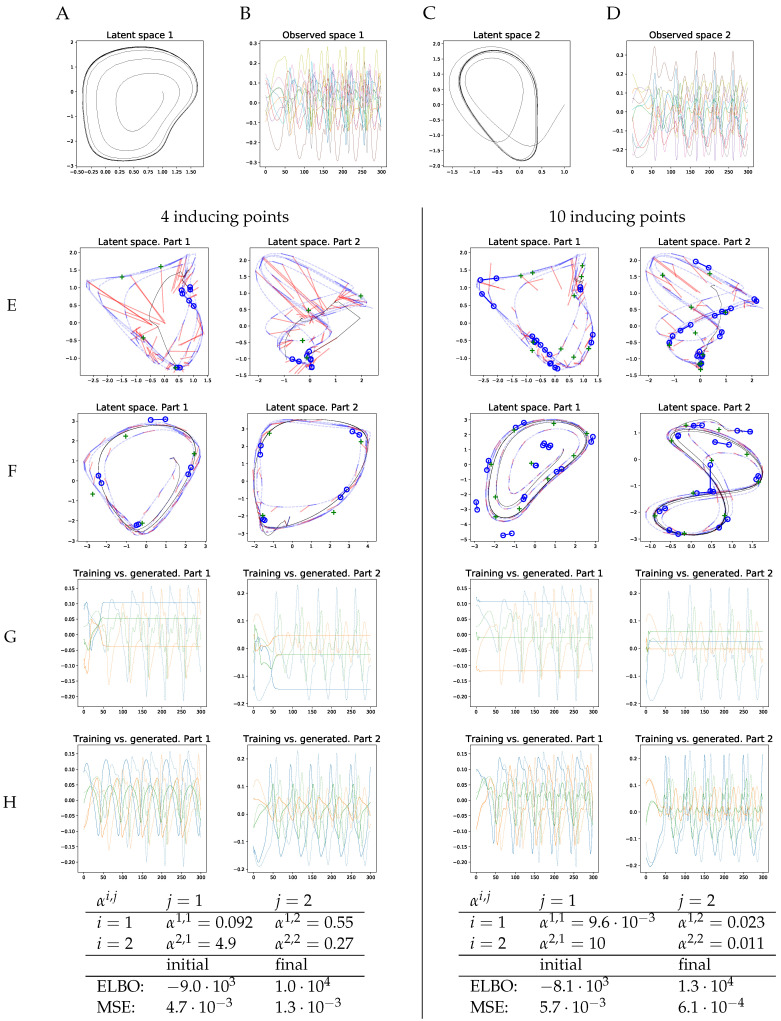
Synthetic training data. (**A**–**D**) Two-dimensional latent dynamics trajectories and corresponding observed 10-dimensional time series. Part 2 is weakly influenced by Part 1, Part 1 is not influenced by Part 2 (see Equation (18)). (**E**) Initial positions of second-order dynamics IPs (connected blue circles) and latent-to-observed IPs (green crosses). Line segments are examples of dynamics mapping inputs (endpoints of blue segments) and values (distal endpoints of red line segments). Black line: mean trajectory, generated by iterating the mean dynamics mapping from the same starting point as in (**F**) Latent space after learning. (**G**) Generated observable time series before learning (solid) and training data (dashed). (**H**) Generated time series (solid) after learning. Only three of the ten observable trajectories are presented for clarity. (**Bottom**) Learned couplings (see Equation (1)); ELBOs and MSEs rounded to two significant digits. Couplings $\alpha^{i,j}$ reflect the dependency structure between parts: Part 1 is not driven by Part 2, but influences Part 2.

We demonstrate the learning of coupled dynamical systems on a synthetic dataset. First, we draw two dynamics transition functions $g^{1},g^{2}\in R^{2}\to R^{2}$ from a GP with an RBF kernel, and then we generate latent trajectories according to:

(18) $$\begin{array}{cc} {\vec{x}}_{t}^{1} & =g^{1}\left({\vec{x}}_{t-1}^{1}\right) \end{array}$$

(19) $$\begin{array}{cc} {\vec{x}}_{t}^{2} & =0.1g^{1}\left({\vec{x}}_{t-1}^{1}\right)+0.9g^{2}\left({\vec{x}}_{t-1}^{2}\right) \end{array}$$

at T=300 timepoints. Thus, we get two first-order, coupled latent dynamical systems, each of dimensionality 2. The trajectory in Latent Space 1 is independent of Latent Space 2, whereas Latent Space 2 is weakly coupled to Latent Space 1. Then, for each of the two parts, we draw 10 observed trajectories from another two RBF GPs with inputs on the latent trajectories. The latent trajectories are shown in Figure 3A,C. Figure 3B,D displays the corresponding observed trajectories. We learned a second-order vCGPDM from these data, iterating the blocked optimization until convergence of the ELBO to machine precision. We chose a second order system for this learning example, because the human movement models in the following are second-order vCGPDMs, too.

The results are shown in Figure 3E–H. Plots on the left half were generated with four IPs, plots in the right half with ten IPs. Figure 3E displays the initial positions of the dynamics IPs (blue circles) at the beginning of learning, connected circles form one second-order IP. Green crosses are the kinematics IPs (latent-to-observed mapping). Initial latent points (dashed blue lines) were obtained from the first two PCA components of the training data. Blue and red line segments are examples of the dynamics mapping: the end-points of the blue segments are the inputs, the distal endpoint of the red segment is the output. As one might expect, the initial conditions do not describe a dynamics which can produce trajectories resembling those in the training data: the black line is the mean latent trajectory, and Figure 3G shows the corresponding observable trajectories.

After learning, the latent trajectories appear to have a limit cycle (black line, Figure 3F), which is required to reproduce the training data. Furthermore, note that the inducing points align with that cycle, and the example mappings (blue and red line segments) indicate clearly how the latent trajectory is formed by iterating the GP mapping. Cross coupling GP mappings omitted for clarity. Note that the vCGPDM with ten IPs can model more complex latent dynamics manifolds than the four-IP vCGPDM. The observable trajectories (Figure 3H) look very similar to the training data up to a small phase shift, particularly for the 10 IP model. This observation is confirmed by the reduced mean squared trajectory error (MSE) between generated and training data after learning, which was evaluated after dynamic time warping [39] of the generated trajectories onto the training data. The MSEs are listed in the table at the bottom of Figure 3, where “final” indicates the values after learning, while “initial” indicates the values at the onset of learning after the latent space trajectories had been initialized to the first two PCA components of the training data. That learning was successful is also indicated by the increased final ELBO, which is higher for the 10 IP model.

We also provided the learned coupling αs in this table. Recall that a low(high) α means a large (small) influence of the corresponding part on the dynamics. The dependency structure between the latent spaces was correctly identified during learning: $a^{2,1}\gg \alpha^{1,1}$, i.e., Part 2 has almost no influence on Part 1. In contrast, $\alpha^{1,2}\approx 2\alpha^{2,2}$, which indicates that Part 1 weakly controls Part 2.

### 5.2. Human Movement Data

Model comparisons and psychophysical tests were carried out on human movement data. We employed a 10-camera PhaseSpace Impulse motion capture system, mapped the resulting position data onto a skeleton with 19 joints and computed joint angles in exponential-map representation, yielding a total of 60 degrees of freedom. Five walking-only and four walking + waving sequences each were used to train the models, as well as ten movements where the human participants were seated and passed a bottle from one hand to the other. Dynamical models were initialized with starting conditions taken from the training data. The blocked optimization was run for at most four iterations, which was enough to ensure convergence. It was terminated earlier if ELBO values did not change within machine precision between two subsequent iterations. Furthermore, we recorded another nine walking sequences for catch trials during the perception experiment, to rule out memorization effects. Generated and recorded sequences were rendered on a neutral avatar. Examples of stimuli, for different numbers of IPs, can be found in the movie example_stimuli.mov in the Supplementary Materials.

### 5.3. Variational Approximations are Better than MAP

**Figure 4:**
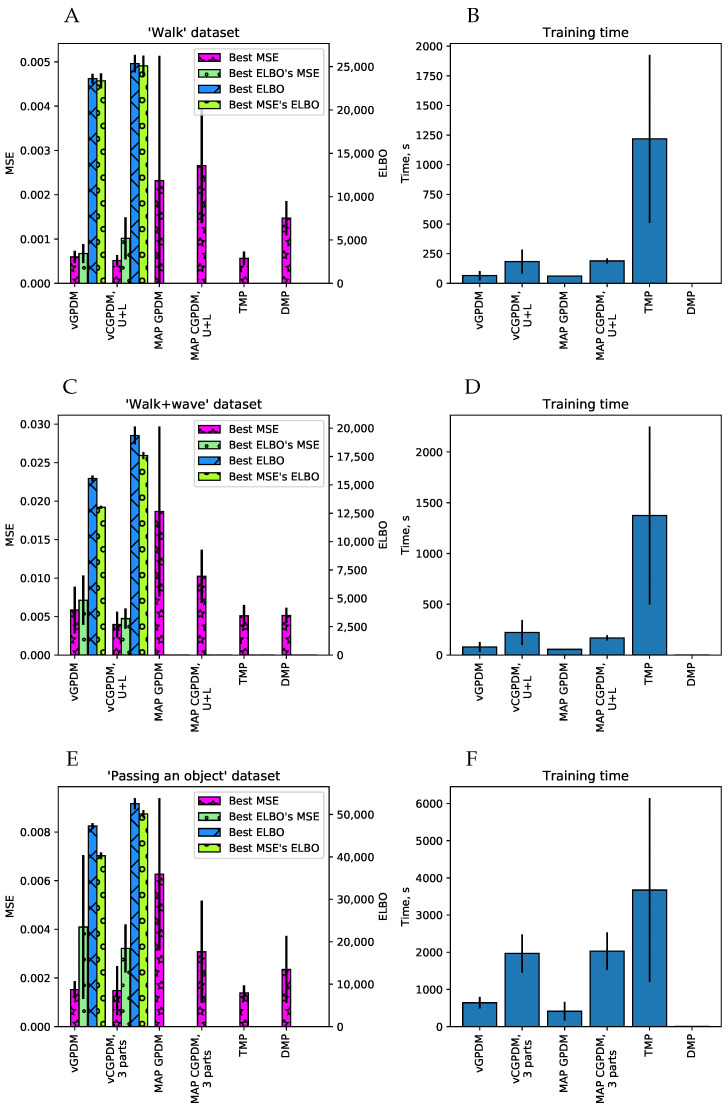
Model comparison results. We plotted the average squared kinematics error on held-out data after dynamic time warping (MSE) and the variational lower bound on the model evidence (ELBO, Equation (A22)), where available, accompanied with corresponding model training time. Error bars are standard errors of the mean. (**A**,**B**) Walking dataset; (**C**,**D**) walking + waving dataset; and (**E**,**F**) “passing a bottle” dataset. Low MSE and high ELBO are better. For details, see text. Figure partially adapted from [19].

We performed cross-validatory model comparison on the following datasets: walking, walking + waving and passing-a-bottle. Examples of these data are shown in the movies in the Supplementary Materials: S1_example_stimuli.mov and S4_pass_the_bottle.mkv. We performed four-, five- and ten-fold crossvalidation, the number of folds was dictated by the dataset size. We were trying determine how the sparsely parameterized vCGPDM performs in comparison to the full CGPDM, and several other MP models from the literature. Held-out data were always one complete trial. Models were trained on the remaining data and the generated trajectory was compared to the held-out one. Cross-validation score was the mean-squared error (MSE) of the kinematics after dynamic time warping [39] of trajectories generated by initializing the model to the first two frames of a held-out trial onto the complete held-out trial. We used dynamic time warping to compensate a slight phase difference in generated motions, which would otherwise lead to a much larger and uninformative MSE. We compared the following models:

- a GPDM with maximum-a-posteriori (MAP) estimation of the latent variables [25], called MAP GPDM in Figure 4;
- a fully marginalized two-part (upper/lower body) CGPDM with MAP estimation of the latent variables [14], called MAP CGPDM U+L;
- a three-part CGPDM model (left hand, right hand, and body) for the non-periodic “passing a bottle” dataset;
- their variational counterparts, vCGPDM 3-part, vCGPDM U+L and vGPDM;
- temporal MPs (TMP, instantaneous linear mixtures of functions of time) [9]; and
- DMPs [12].

All latent spaces were three-dimensional. We tried 4–30 latent-to-observed IPs and 2–30 dynamics IPs. The MSE optima were near 10–15 IPs for both the walking and the walking + waving datasets, and near eight IPs for the “passing a bottle”. MAP GPDM and MAP CGPDM learning do not use any approximations or inducing points; they are the full GPs with covariance matrices $K\in R^{T*T}$.

For the TMPs, we used up to 10 primitives; the MSE optimum was located at approximately six. For the DMPs, we used between 1 and 50 basis functions, and the lowest MSE was found around 15.

The results are plotted in Figure 4. Generally, the walking + waving movement is more difficult to reproduce for all models than walking only: the MSE of the latter is lower than that of the former, and the ELBO is higher. This indicates that the latter is a more complex movement, see also the movie modular_primitives.avi in the online Supplementary Materials. The two-part vCGPDM reaches the lowest MSE compared to all other models. Clearly, it is better than the full-capacity (no IPs) MAP models, which means that the extra effort of developing of a variational approximation which explicitly represents an approximation to the latent states’ posterior and needs to store only ≈10 IPs rather than ≈10${}^{4}$ data points was well spent. In addition, the best ELBO’s MSE (that is, the MSE at the maximum of the ELBO) is a fairly good predictor of the best MSE, which justifies our simple variational approximation for model selection.

The vCGPDM U+L outperforms the vGPDM particularly on the “walking + waving” dataset. This shows the usefulness of having modular, coupled dynamics models when the (inter)acting (body)parts execute partially independent movements.

The vCGPDM with three parts for “passing a bottle” does not show a clear advantage over the monolithic model in the cross-validation test, and is on par with the TMP model. However, dynamics factorization did not affect the performance either. This may be indicative for the strong coupling necessary to successfully pass an object from one hand to the other. Such a strong coupling is parsimoniously expressed by having a single latent dynamical system drive all observable degrees of freedom.

The timing results show that the training times of the vCGPDM are usually less than 15 min. Error bars are standard deviations, estimated across all numbers of IPs and cross-validation splits. The rather large training time for the TMP model is due to the implementation from [9] which optimizes a rather large covariance matrix between all MPs.

### 5.4. A Small Number of IPs Yields Perceptually Convincing Movements

We conducted a psychophysical experiment to quantify the perceptual validity of the generated movements. More specifically, we investigated the model complexity required for perceptually convincing movements.

**Figure 5:**
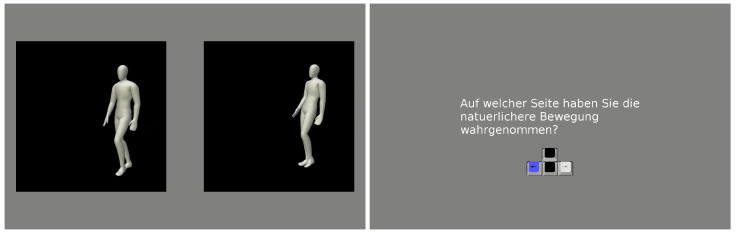
Psychophysical Experiment. In each trial, a natural and a generated movement were simultaneously presented to participants (**left**). After presentation, they used the arrow keys to choose the movement perceived as more natural (**right**). There was no time limit on the response, but typically participants responded quickly (less than 1 s). After the response, people were asked to fixate a cross in the middle of the screen, which appeared for a second. The length of the stimuli was 1.8 s, with a total of 1170 presentations. A video of the experiment called S2_experiment_demo.avi is provided in the Supplementary Materials.

**Experiment**: Thirty-one human observers (10 male, mean age: 23.8±3.5a) participated in a two-alternative forced-choice task to distinguish between natural and generated movements (see Figure 5 for an example of an experimental trial). Natural movements consisted of 15 walking movements. The artificial movements were generated by a two-part (upper/lower body) vCGPDM. We used 2–16 dynamics IPs and 4–16 latent-to-observed IPs. We chose these numbers based on pilot tests to span the range from clearly unnatural to very natural looking movements. To test whether participants simply memorized the 15 natural stimuli during the experiment, we added 10 catch trials in the last quarter of the experiment where previously unused natural movements were tested against the known natural stimuli. The trial sequence was randomized for every subject. All experimental procedures were approved by the local ethics commission.

**Figure 6:**
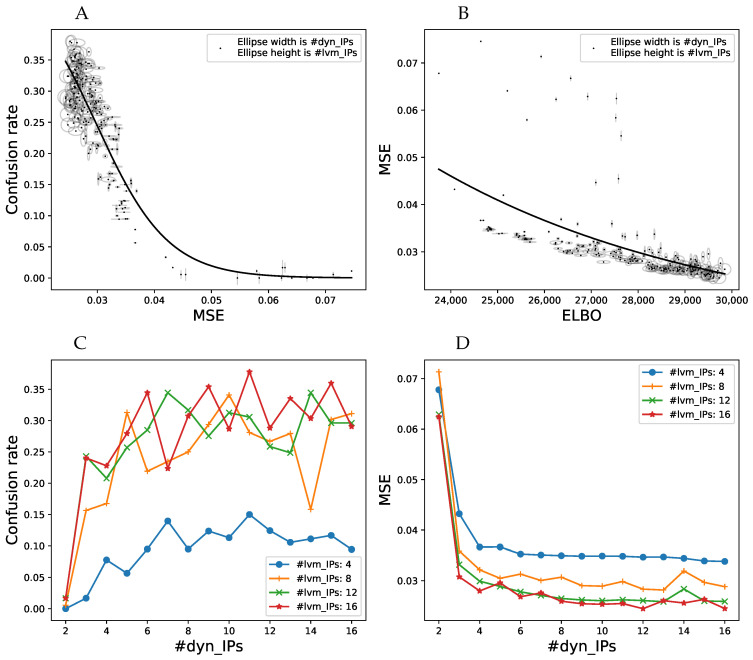
(**A**) Confusion rate between natural and vCGPDM-generated stimuli as a function of mean-squared residual error (MSE) on the training data, averaged across all participants. Each data point represents one combination between number of IPs/IVs for the latent-to-observed mapping (indicated by ellipse height) and number of IPs/IVs for the dynamics mappings (ellipse widths). A confusion rate of 0.5 indicates that human observers are not able to distinguish replays of real movements from model-generated counterparts. The vCGPDM is approaching this limit from below for a fairly small number of IPs/IVs. Solid line: fit with logistic sigmoid function. (**B**) Relationship between training MSE and ELBO. Solid line: fit with exponential function. Additional dynamics IPs contribute more to the reduction of the MSE than latent-to-observed IPs. MSE and therefore confusion rate can be predicted well from ELBO if ELBO > 28,500. (**C**,**D**) Influence of number of dynamics IPs on the confusion rate and MSE, respectively, for a selected number of latent-to-observed IPs. The confusion rate has a broad maximum around 8–12 dynamics IPs, whereas the MSE has a shallow minimum at that location.

**Results**: We computed the confusion rate, i.e., the frequency of choosing the model-generated movement as more natural across all participants as a function of the number of IPs for the dynamics and latent-to-observed mappings. Optimally, we might expect this rate to approach $\frac{1}{2}$ when the generated movements are indistinguishable from the natural ones. We investigated if the confusion rate approached this limit, how it depends on the mean-squared residual error on the training data, and how this error is connected to the ELBO. The results are plotted in Figure 6. We also fitted the confusion rate data with a logistic sigmoid $\frac{0.5}{1+\exp(a\cdot MSE+c)}$ (solid line in Figure 6A), and the MSE with an exponential function (solid line in Figure 6, right). Each data point represents one combination of dynamics/latent-to-observed IP numbers, indicated by width and height of the ellipses. Clearly, confusion rate increases fairly monotonically with decreasing MSE, as indicated by the good logistic sigmoid fit. Furthermore, models with more IPs also tend to yield higher confusion rates. A sufficient number (>10) dynamics IPs is more important than a large number of latent-to-observed IPs, which can be seen by the very narrow ellipses in the region with high MSE, and many wider ellipses in the lower MSE part of the figure. A similar observation can be made about the relationship between ELBO and MSE (Figure 6B). It indicates that ELBO is already a good predictor for the model performance. For a very small number of dynamics IPs, increasing the number of latent-to-observed IPs does not decrease the MSE as much as increasing the dynamics IPs does. Moreover, note that the relationship between MSE and ELBO becomes fairly monotonic when ELBO > 28,500, which is where human perceptual performance can be predicted from ELBO. While the confusion rate has not quite reached its theoretical maximum in our experiment, these results are evidence that human perceptual expectations can be nearly met with very compactly parameterized MP models (Figure 6C,D). Moreover, good dynamics models seem to outweigh precise kinematics. We found no evidence for stimulus memorization from the confusion rates of the catch trials.

### 5.5. Modularity Test

**Figure 7:**
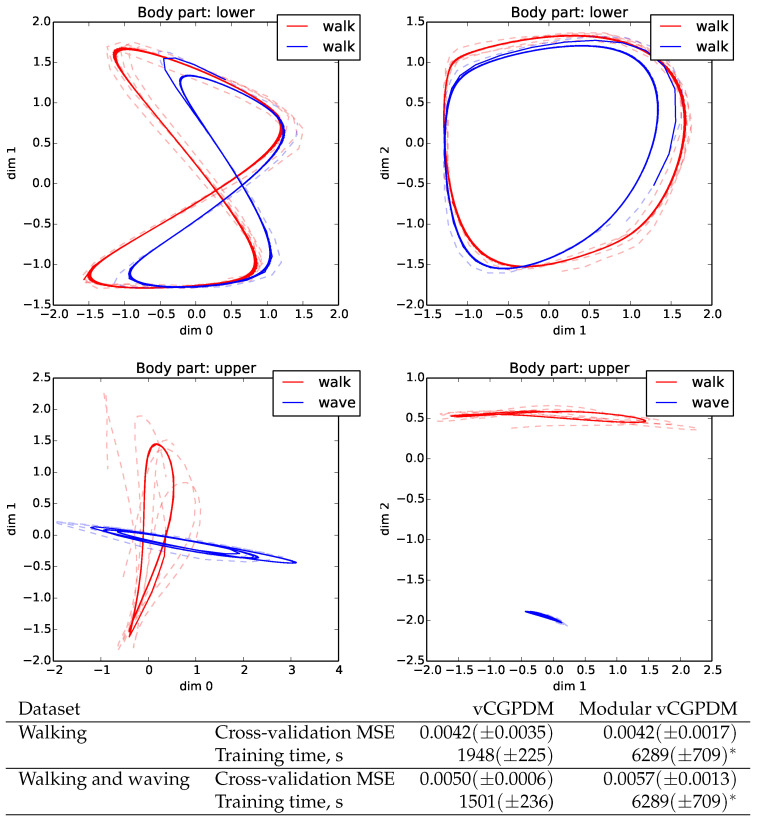
(**Top**) Modularity example. Shown are 2D projections of generated 3D latent space trajectories (solid) and training data (dashed). Blue: walk + wave movements; red: walk + normal arm swing. Dynamics IPs re-used across movements for lower body. (**Bottom**) Cross-validation MSEs of non-modular and modular vCGPDM. Modular vCGPDM was trained on the combined dataset; training time (*) is shared between both movement datasets.

Next, we examined if the intended modularization of our model can be used to compose novel movements from previously learned parts. We trained a vCGPDM consisting of one part for the lower body (below and including pelvis), and a second part for the upper body. Twenty-five IPs for the latent-to-observed mapping of each part were shared across all movements. The walking MP, parameterized by 16 IPs for the lower-body dynamics and the lower-to-upper mappings, was also shared. We used a different set of 16 IPs for the upper body MPs between arm-swing and waving. Furthermore, the coupling $\alpha^{j,i}$ were learned anew for each combination of upper/lower MPs. The resulting latent space trajectories are plotted in Figure 7. All generated trajectories (solid lines) are on average close to the training data (dashed lines). While the walking trajectories for the lower body are very similar for the two movements, the upper body trajectories clearly differ. Movements generated from this model are very natural (see video S3_modular_primitives.mov in the Supplementary Materials). This is a first demonstration that the vCGPDM with non-marginalized couplings can be used to learn a library of compactly parameterized MPs, from which novel movements can be produced with little additional memory requirements (i.e., new coupling $\alpha^{j,i}$ only).

For a quantitative evaluation, we looked at the leave-one-out cross-validation MSEs of the vCGPDM trained on datasets separately and modular vCGPDM trained on both datasets (see Figure 7, bottom). Within the standard errors, MSEs are equal, indicating that modular re-use of previously trained components does not necessarily sacrifice accuracy, while reducing storage requirements. Training time for the modular vCGPDM is larger due to the learning of the combined dataset and optimizing the couplings afterwards. This time would be amortized if more compositional movements were learned, where previously learned parts could be reused.

## 6. Conclusions

The vCGPDM, a full variational approximation to the CGPDM, allows for learning a deterministic approximation of latent space trajectories, and compactly parameterizing dynamics and kinematics mappings. First, we showed that the sparsely parameterized vCGPDM outperforms the full-capacity, monolithic CGPDM employing MAP to approximate the latent dynamics posterior. It also surpasses other current MP models; we speculate that this is accomplished by its learnable dynamics.

Second, we demonstrated that our compact representation of the latent space dynamics, and of the latent-to-observed mapping, enables the model to generate perceptually convincing full-body movements with a fairly small number of IPs To our knowledge, a systematic investigation of the number of IPs needed for perceptual plausibility had not been done before, albeit more monolithic models were in the focus of earlier studies [27,34,40]. Moreover, we demonstrated that a high enough ELBO can be used to predict average human classification performance, which might allow for an automatic model selection process when training the model on large databases. Within the range of IPs which we tested, the ELBO was still increasing with their number. We chose that range because we wanted to see how few IPs would still lead to perceptually indistinguishable movements. Due to experimental time constraints, we did not investigate perceptual performance at the point where the ELBO begins to decrease with increasing IPs (i.e., the approximately optimal model), but we plan to do that in the future.

Third, we showed that the model can be employed in a modular fashion, using one lower-body dynamics model, and coupling it to two different models for the upper body. Note that the lower-to-upper coupling function was the same for the two upper-body models. Each of these models, including the coupling functions to the other model parts, may therefore be viewed as a modular MP that is parameterized compactly by a small number of IPs and values. This sparse parameterization allows us to infer modular MPs from a large collection of movements, and investigate their composition. To generate complex movement sequences, we will put a switching prior on top of the dynamical models, as in [29].

We are currently researching sensorimotor primitives, i.e., MPs that can be used to predict sensory input and be controlled by it via conditioning. This conditioning can take place on at least two timescales: a short one (while the MP is running), thus effectively turning the MPs into flexible control policies, such the probabilistic MPs described by Paraschos et al. [41], and a long timescale, i.e., the planning of the movement. This could be implemented by learning a mapping from goals and affordances onto the coupling weights, comparable to the DMPs with associative skill memories [42]. There is evidence that humans modulate the coupling between their MPs during the planning stage: whole-body posture changes have been observed in anticipation of reaching for a goal object in a known location, even if the object is currently invisible [43].

Lastly, we note that our CGPDM could be used as a flexible policy model for PILCO-style reinforcement learning (Probabilistic Inference for Learning Control, [44]). PILCO requires a dynamics model that can propagate uncertainties through time; the vCGPDM is able to do that. Thus, our model could be used as a lower dimensional dynamics model which can capture the dependencies between observable variables via latent space uncertainties.

## Figures and Tables

**Table 1:** Overview of movement primitive models compared in this paper. (v)CGPDM, (variational) coupled Gaussian process dynamical model; (v)GPDM, (variational) Gaussian process dynamical model; TMP, temporal movement primitives; DMP, dynamical movement primitives. Modular, learns reusable MPs. Scalable, below cubic learning complexity with respect to the data set size; Compact, size of the effective parameterization does not grow with the data set size; Canonical dynamics, dynamics model specified before learning; Learned dynamics, dynamics model is a free-form function.

	Modular	Scalable	Compact	Canonical Dynamics	Learned Dynamics
vCGPDM	✓	✓	✓	✗	✓
CGPDM	✗	✗	✗	✗	✓
vGPDM	✗	✓	✓	✗	✓
GPDM	✗	✗	✗	✗	✓
TMP	✓	✓	✓	✗	✗
DMP	✓	✓	✓	✓	✗

## Supplementary Materials

The following are available online http://www.mdpi.com/1099-4300/20/10/724/s1, Video S1: example_stimuli, Video S2: experiment_demo, Video S3: modular_primitives, Video S4: pass_the_bottle.

## Abbreviations

The following abbreviations are used in this manuscript:

MP	movement primitive
DMP	dynamical movement primitive
GP	Gaussian process
GPDM	Gaussian process dynamical model
CGPDM	coupled Gaussian process dynamical model
vCGPDM	variational coupled Gaussian process dynamical model
IP	inducing point
IV	inducing value
MSE	mean squared error

## Appendix A. Exact Variational Optimization of Parts of the ELBO

While optimizing the full variational posterior in augmented Gaussian processes models with respect to the IVs, the following type of term appears several times in the ELBO equation:

(A1) $$\begin{array}{cc} R(q\left(\vec{u}\right),r\left(\vec{v}\right)) & =\int q\left(\vec{u}\right)\left(f\left(r\left(\vec{v}\right),\vec{u}\right)+\log\frac{p(\vec{u})}{q(\vec{u})}\right)d\vec{u}\\ & =\int q\left(\vec{u}\right)f\left(r\left(\vec{v}\right),\vec{u}\right)d\vec{u}+\int q\left(\vec{u}\right)\log p\left(\vec{u}\right)d\vec{u}-\int q\left(\vec{u}\right)\log q\left(\vec{u}\right)d\vec{u} \end{array}$$

To simplify the optimization of such terms, we would like to carry out the optimization with respect to the density $q(\vec{u})$ analytically to remove the dependency on $q(\vec{u})$. Note that we allow only $q\left(\vec{u}\right),r\left(\vec{v}\right)$ to vary, while the functions $f(r\left(\vec{v}\right),\vec{u})$ and $p(\vec{u})$ are assumed to be fixed. To this end, we calculate for the optimal variational $q^{*}\left(\vec{u}\right)$ in the above equation. This approach was suggested in [23], however, it is not well described there. Here, we give an extended derivation. A necessary condition for maximality is a vanishing functional derivative under the constraint that the density $q(\vec{u})$ is normalized to one:

(A2) $$\int q\left(\vec{u}\right)d\vec{u}-1=0$$

which is fulfilled at the stationary points of the Lagrangian

(A3) $$X\left(q\left(\vec{u}\right),r\left(\vec{v}\right)\right)=R\left(q\left(\vec{u}\right),r\left(\vec{v}\right)\right)+\lambda \left(\int q\left(\vec{u}\right)d\vec{u}-1\right)$$

where λ is chosen so that Equation (A2) holds. Taking the functional derivative of $X(q\left(\vec{u}\right),q\left(\vec{v}\right))$ and setting it to zero yields

(A4) $$\begin{array}{cc} \frac{\delta X(q\left(\vec{u}\right),q\left(\vec{v}\right))}{\delta q(\vec{u})} & =f\left(r\left(\vec{v}\right),\vec{u}\right)+\log p\left(\vec{u}\right)-\log q\left(\vec{u}\right)-1+\lambda =0 \end{array}$$

and therefore, denoting $Z=\exp\left(-\lambda +1\right)$

(A5) $$\begin{array}{cc} q^{*}\left(\vec{u}\right) & =\exp(f\left(r\left(\vec{v}\right),\vec{u}\right)+\log p\left(\vec{u}\right)-1+\lambda ) \end{array}$$

(A6) $$\begin{array}{cc} q^{*}\left(\vec{u}\right) & =\frac{1}{Z}p\left(\vec{u}\right)\exp\left(f\left(r\left(\vec{v}\right),\vec{u}\right)\right) \end{array}$$

(A7) $$\begin{array}{cc} Z & =\exp\left(-\lambda +1\right)=\int p\left(\vec{u}\right)\exp\left(f\left(r\left(\vec{v}\right),\vec{u}\right)\right)d\vec{u} \end{array}$$

Substituting the optimal $q^{*}\left(\vec{u}\right)$ into the original term, we get:

(A8) $$\begin{array}{cc} R(r\left(\vec{v}\right)) & =\int \frac{1}{Z}p\left(\vec{u}\right)\exp\left(f\left(r\left(\vec{v}\right),\vec{u}\right)\right)\left(f\left(r\left(\vec{v}\right),\vec{u}\right)+\log\frac{p(\vec{u})}{\frac{1}{Z}p\left(\vec{u}\right)\exp\left(f\left(r\left(\vec{v}\right),\vec{u}\right)\right)}\right)d\vec{u}\\ & =\int \frac{1}{Z}p\left(\vec{u}\right)\exp\left(f\left(r\left(\vec{v}\right),\vec{u}\right)\right)\left(\log\frac{p\left(\vec{u}\right)\exp\left(f\left(r\left(\vec{v}\right),\vec{u}\right)\right)}{\frac{1}{Z}p\left(\vec{u}\right)\exp\left(f\left(r\left(\vec{v}\right),\vec{u}\right)\right)}\right)d\vec{u}\\ & =\log\left(Z\right)\frac{1}{Z}\int p\left(\vec{u}\right)\exp\left(f\left(r\left(\vec{v}\right),\vec{u}\right)\right)d\vec{u}\\ & =\log\int p\left(\vec{u}\right)\exp\left(f\left(r\left(\vec{v}\right),\vec{u}\right)\right)d\vec{u} \end{array}$$

This is the optimized version of Equation (A1), which depends only on $r(\vec{v})$.

## Appendix B. ARD RBF Kernel Ψ Statistics. Full Covariance Variational Parameters Case.

During the computation of the ELBO, it is necessary to evaluate expected values of Gaussian process kernel functions under the variational posterior distributions. Here, we derive these expectations, referred to as Ψ statistics in the literature [16], for the type of kernel we used in this paper: an automatic relevance determination, squared exponential kernel (ARD RBF). The ARD RBF kernel is defined as:

(A9) $$\begin{array}{cc} k(\vec{x},{\vec{x}}^{\prime }) & =\sigma_{f}^{2}\exp\left(-\frac{1}{2}\sum_{q=1}^{Q}\frac{{\left({\left(\vec{x}\right)}_{q}-{\left(\vec{x}\right)}_{q}^{\prime }\right)}^{2}}{\lambda_{q}}\right) \end{array}$$

where $\lambda_{q}$ are the ARD factors, $\sigma_{f}^{2}$ is the variance of the kernel and *Q* is the dimensionality of $\vec{x}$. In matrix notation:

(A10) $$\begin{array}{cc} \lambda & =\mathrm{diag}(\lambda_{1}\ldots \lambda_{Q}) \end{array}$$

(A11) $$\begin{array}{cc} k(\vec{x},{\vec{x}}^{\prime }) & =\sigma_{f}^{2}\exp\left(-\frac{1}{2}{\left(\vec{x}-{\vec{x}}^{\prime }\right)}^{T}\lambda^{-1}\left(\vec{x}-{\vec{x}}^{\prime }\right)\right) \end{array}$$

Let $\vec{x}$ be a random variable drawn from multivariate Gaussian distributions with mean $\vec{\mu }$ and covariance matrix *S*. Consider the following form of the approximate variational posterior distribution of $\vec{x}$:

(A12) $$\begin{array}{cc} q(\vec{x}) & =N({\vec{x}}_{n}|\vec{\mu },S) \end{array}$$

The $\Psi_{0}$ statistic is the expectation of the kernel for two identical arguments, which is easy to calculate:

(A13) $$\begin{array}{cc} \Psi_{0} & =\int k\left({\vec{x}}_{n},{\vec{x}}_{n}\right)N\left({\vec{x}}_{n}|{\vec{\mu }}_{n},S_{n}\right)d{\vec{x}}_{n}\\ & =\int \sigma_{f}^{2}N\left({\vec{x}}_{n}|{\vec{\mu }}_{n},S_{n}\right)d{\vec{x}}_{n}\\ & =\sigma_{f}^{2}\int N\left({\vec{x}}_{n}|{\vec{\mu }}_{n},S_{n}\right)d{\vec{x}}_{n}\\ & =\sigma_{f}^{2} \end{array}$$

The $\Psi_{1}$ statistic is the expectation with respect to one kernel argument, given that the other is constant:

(A14) $$\begin{array}{cc} \Psi_{1} & =\int k\left(\vec{x},\vec{z}\right)N\left(\vec{x}|\vec{\mu },S\right)d\vec{x}\\ & =\int \sigma_{f}^{2}\exp\left(-\frac{1}{2}{\left(\vec{x}-\vec{z}\right)}^{T}\lambda^{-1}\left(\vec{x}-\vec{z}\right)\right)N\left(\vec{x}|\vec{\mu },S\right)d\vec{x} \end{array}$$

To evaluate the integral, complete the ARD RBF kernel to a scaled Gaussian distribution with covariance matrix λ and mean $\vec{z}$:

(A15) $$\begin{array}{cc} \Psi_{1} & =\sigma_{f}^{2}\int \frac{Z(\lambda )}{Z(\lambda )}\exp\left(-\frac{1}{2}{\left({\vec{x}}_{r}-\vec{z}\right)}^{T}\lambda^{-1}\left({\vec{x}}_{r}-\vec{z}\right)\right)N\left({\vec{x}}_{n}|{\vec{\mu }}_{n},S_{n}\right)d\vec{x}\\ & =\sigma_{f}^{2}\;Z\left(\lambda \right)\int N\left(\vec{x}|\vec{z},\lambda \right)N\left(\vec{x}|\vec{\mu },S\right)d\vec{x} \end{array}$$

with $Z\left(\lambda \right)={\left(2\pi \right)}^{Q/2}\sqrt{\left|\lambda \right|}$. The integral over the product of two Gaussians can be carried out to yield (see [45], Identity 371):

(A16) $$\begin{array}{cc} \Psi_{1} & =\sigma_{f}^{2}\;Z\left(\lambda \right)N\left(\vec{z}|\vec{\mu },\lambda +S\right)\\ & =\sigma_{f}^{2}\;{\left(2\pi \right)}^{Q/2}\sqrt{\left|\lambda \right|}\frac{1}{{\left(2\pi \right)}^{Q/2}\sqrt{\left|\lambda +S\right|}}\exp\left(-\frac{1}{2}{\left(\vec{z}-\vec{\mu }\right)}^{T}{\left(\lambda +S\right)}^{-1}\left(\vec{z}-\vec{\mu }\right)\right)\\ & =\sigma_{f}^{2}\;\frac{\sqrt{\left|\lambda \right|}}{\sqrt{\left|\lambda +S\right|}}\exp\left(-\frac{1}{2}{\left(\vec{z}-\vec{\mu }\right)}^{T}{\left(\lambda +S\right)}^{-1}\left(\vec{z}-\vec{\mu }\right)\right)\\ & =\sigma_{f}^{2}\;\sqrt{\frac{\prod_{q=1}^{Q}\lambda_{q}}{\left|\lambda +S\right|}}\exp\left(-\frac{1}{2}{\left(\vec{z}-\vec{\mu }\right)}^{T}{\left(\lambda +S\right)}^{-1}\left(\vec{z}-\vec{\mu }\right)\right) \end{array}$$

The $\Psi_{2}$ statistic integral, which correlates two kernel function values at different points $\vec{z}$ and ${\vec{z}}^{\prime }$, can be solved in a similar manner: first, by collecting terms in the exponents and completing quadratic forms, and second, by the application of Identity 371 from [45]:

(A17) $$\begin{array}{cc} \Psi_{2} & =\int k\left(\vec{x},\vec{z}\right)k\left({\vec{z}}^{\prime },\vec{x}\right)N\left(\vec{x}|\vec{\mu },S\right)d\vec{x}\\ & ={\left(\sigma_{f}^{2}\;Z\left(\lambda \right)\right)}^{2}\;\int N\left(\vec{x}|\vec{z},\lambda \right)N\left(\vec{x}|{\vec{z}}^{\prime },\lambda \right)N\left(\vec{x}|\vec{\mu },S\right)d\vec{x}\\ & ={\left(\sigma_{f}^{2}Z\left(\lambda \right)\right)}^{2}\;\int N\left({\vec{z}}^{\prime }|\vec{z},2\lambda \right)N\left(\vec{x}|\frac{1}{2}\left({\vec{z}}^{\prime }+\vec{z}\right),\frac{1}{2}\lambda \right)N\left(\vec{x}|\vec{\mu },S\right)d\vec{x}\\ & ={\left(\sigma_{f}^{2}Z\left(\lambda \right)\right)}^{2}\;N\left({\vec{z}}^{\prime }|\vec{z},2\lambda \right)\int N\left(\vec{x}|\frac{1}{2}\left(\vec{z}+{\vec{z}}^{\prime }\right),\frac{\lambda }{2}\right)N\left(\vec{x}|\vec{\mu },S\right)d\vec{x}\\ & ={\left(\sigma_{f}^{2}Z\left(\lambda \right)\right)}^{2}\;N\left({\vec{z}}^{\prime }|\vec{z},2\lambda \right)N\left(\vec{\mu }|\frac{1}{2}\left(\vec{z}+{\vec{z}}^{\prime }\right),\frac{\lambda }{2}+S\right)\\ & =\sigma_{f}^{4}{\left(2\pi \right)}^{Q}\left(\prod_{q=1}^{Q}\lambda_{q}\right)\;N\left({\vec{z}}^{\prime }|\vec{z},2\lambda \right)\;N\left(\vec{\mu }|\frac{\vec{z}+{\vec{z}}^{\prime }}{2},\frac{\lambda }{2}+S\right) \end{array}$$

For the case of a diagonal covariance matrix *S* the $\Psi_{2}$ statistic can be simplified further [16].

## Appendix C. vCGPDM Dynamics ELBO Derivation

We now present a detailed derivation of the ELBO with a focus on the dynamics component. Assume we deal with *M* parts. We have M×M latent dynamics mappings, which are combined into *M* mappings with product of experts—multiplying and renormalizing the distributions from all parts’ predictions about each part. Each of the M×M mappings $f^{j,i}\left(\right)$ is augmented with IPs ${\vec{z}}_{:}^{j,i}$ and IVs ${\vec{u}}_{:}^{j,i}$, which are drawn out of the same GP priors as the mappings. For clarity, we omit spelling out the dependence of the IVs on the IPs in the following, and we ask the reader to remember that any distribution over IVs is implicitly conditioned onto the corresponding IPs. The full augmented joint distribution of the model, which is derived in Section 4 (Equation (5)), is:

(A18) $$\begin{array}{cc} p({\vec{y}}_{:}^{:},{\vec{g}}_{:}^{:},{\vec{x}}_{:}^{:},{\vec{v}}_{:}^{:},{\vec{f}}_{:}^{:,:},{\vec{u}}_{:}^{:,:}) & =p\left({\vec{y}}_{:}^{:}|{\vec{g}}_{:}^{:}\right)p\left({\vec{g}}_{:}^{:}|{\vec{x}}_{:}^{:},{\vec{v}}_{:}^{:}\right)p\left({\vec{v}}_{:}^{:}\right)p\left({\vec{x}}_{:}^{:},{\vec{f}}_{:}^{:,:}|{\vec{u}}_{:}^{:,:}\right)p\left({\vec{u}}_{:}^{:,:}\right)\\ & =\left[\prod_{i=1}^{M}p\left({\vec{y}}_{:}^{i}|{\vec{g}}_{:}^{i}\right)p\left({\vec{g}}_{:}^{i}|{\vec{x}}_{:}^{i},{\vec{v}}_{:}^{i}\right)p\left({\vec{v}}_{:}^{i}\right)\right]p\left({\vec{x}}_{:}^{:},{\vec{f}}_{:}^{:,:}|{\vec{u}}_{:}^{:,:}\right)p\left({\vec{u}}_{:}^{:,:}\right)\\ & =\left[\prod_{i=1}^{M}\left[\prod_{d=1}^{D_{i}}p\left({\left({\vec{y}}_{:}^{i}\right)}_{d}|{\left({\vec{g}}_{:}^{i}\right)}_{d}\right)p\left({\left({\vec{g}}_{:}^{i}\right)}_{d}|{\vec{x}}_{:}^{i},{\left({\vec{v}}_{:}^{i}\right)}_{d}\right)p\left({\left({\vec{v}}_{:}^{i}\right)}_{d}\right)\right]\right]\\ & \times \left[\prod_{t=1}^{T}\left[\prod_{i=1}^{M}p\left({\vec{x}}_{t}^{i}|{\vec{f}}_{t}^{:,i},\alpha^{:,i}\right)\right]\left[\prod_{i=1}^{M}\prod_{j=1}^{M}p\left({\vec{f}}_{t}^{j,i}|{\vec{f}}_{1:t-1}^{j,i},{\vec{x}}_{0:t-1}^{j},{\vec{u}}_{:}^{j,i}\right)\right]\right]\\ & \times \left[\prod_{i=1}^{M}\prod_{j=1}^{M}p\left({\vec{u}}_{:}^{j,i}\right)\right]\left[\prod_{i=1}^{M}p\left({\vec{x}}_{0}^{i}\right)\right] \end{array}$$

The full proposal variational posterior is (Equation (12) in Section 4):

(A19) $$\begin{array}{cc} q({\vec{g}}_{:}^{:},{\vec{x}}_{:}^{:},{\vec{v}}_{:}^{:},{\vec{f}}_{:}^{:,:},{\vec{u}}_{:}^{:,:}) & =p\left({\vec{g}}_{:}^{:}|{\vec{x}}_{:}^{:},{\vec{v}}_{:}^{:}\right)q\left({\vec{v}}_{:}^{:}\right)p\left({\vec{f}}_{:}^{:,:}|{\vec{x}}_{:}^{:},{\vec{u}}_{:}^{:,:}\right)q\left({\vec{x}}_{:}^{:}\right)q\left({\vec{u}}_{:}^{:,:}\right)\\ & =p\left({\vec{g}}_{:}^{:}|{\vec{x}}_{:}^{:},{\vec{v}}_{:}^{:}\right)q\left({\vec{v}}_{:}^{:}\right)\left[\prod_{t=1}^{T}\prod_{i=1}^{M}\prod_{j=1}^{M}p\left({\vec{f}}_{t}^{j,i}|{\vec{f}}_{1:t-1}^{j,i},{\vec{x}}_{0:t-1}^{j},{\vec{u}}_{:}^{j,i}\right)\right]q\left({\vec{x}}_{:}^{:}\right)q\left({\vec{u}}_{:}^{:,:}\right) \end{array}$$

Thus, the ELBO is given by:

(A20)L(Θ)=∫dg→::dx→::dv→::df→::,:du→::,:q(g→::,x→::,v→::,f→::,:,u→::,:)logp(y→::,g→::,x→::,v→::,f→::,:,u→::,:)q(g→::,x→::,v→::,f→::,:,u→::,:)=∑i=1MLkini+Ldyn(A21)=∑i=1M∑d=1D∫dx→:idv→:id(g→:i)dp((g→:i)d|x→:i,v→:i)q(x→:i)q(v→:i)logp((y→:i)d|(g→:i)d)q(v→:i)+∫du→::,:q(u→::,:)∑t=1T∫q(x→t:)q(x→−t:)∫df→t:,:∏i=1M∏j=1Mp(f→tj,i|f→1:t−1j,i,x→0:t−1j,u→:j,i)log∏i=1Mp(x→ti|f→t:,i,α:,i)(A22)+∫du→::,:q(u→::,:)logp(u→::,:)q(u→::,:)+∑t=01∫q(x→t:)logp(x→t:)dx→t:+H(q(x→::))

The term in Equation (A21), which we call $\sum_{i=1}^{M}L_{kin}^{i}$, is the GPLVM ELBO up to $H(q\left({\vec{x}}_{:}^{:}\right))$ and is given in [16]. Next, we consider only the ELBO component which is relevant for the dynamics $L_{dyn}$ (last two lines of the right hand side of Equation (A22)) and apply the sufficient statistics assumption: knowing ${\vec{x}}_{-t}^{j}$ and ${\vec{u}}_{:}^{j,i}$ is sufficient for the ${\vec{f}}_{t}^{j,i}$ distribution, i.e., $p\left({\vec{f}}_{t}^{j,i}|{\vec{f}}_{1:t-1}^{j,i},{\vec{x}}_{0:t-1}^{j},{\vec{u}}_{:}^{j,i}\right)=p\left({\vec{f}}_{t}^{j,i}|{\vec{x}}_{0:t-1}^{j},{\vec{u}}_{:}^{j,i}\right)$. This assumption lower-bounds $L_{dyn}$, because it constrains the variational posterior in Equation (A19) away from the correct solution. The sum over the initial latent points in the last line of Equation (A22) may be longer or shorter depending on the dynamics model order, here we use a second order model. The innermost integral can then be written as:

(A23) $$\begin{array}{cc} A & =\int \left[\prod_{i=1}^{M}\prod_{j=1}^{M}p\left({\vec{f}}_{t}^{j,i}|{\vec{x}}_{-t}^{j},{\vec{u}}_{:}^{j,i}\right)\right]\log\prod_{i=1}^{M}p\left({\vec{x}}_{t}^{i}|{\vec{f}}_{t}^{:,i},\alpha^{:,i}\right)d{\vec{f}}_{t}^{:,:}\\ & =\sum_{i=1}^{M}\int \left[\prod_{j=1}^{M}p\left({\vec{f}}_{t}^{j,i}|{\vec{x}}_{-t}^{j},{\vec{u}}_{:}^{j,i}\right)\right]\log p\left({\vec{x}}_{t}^{i}|{\vec{f}}_{t}^{:,i},\alpha^{:,i}\right)d{\vec{f}}_{t}^{:,i}\\ & =\sum_{i=1}^{M}\int \left[\prod_{j=1}^{M}N\left({\vec{f}}_{t}^{j,i}|{\vec{\mu }}_{{\vec{f}}_{t}^{j,i}},S_{{\vec{f}}_{t}^{j,i}}\right)\right]\log N\left({\vec{x}}_{t}^{i}|\alpha^{i}\sum_{j=1}^{M}{\left(\alpha^{j,i}\right)}^{-1}{\vec{f}}_{t}^{j,i},I\alpha^{i}\right)d{\vec{f}}_{t}^{:,i}\\ & =\sum_{i=1}^{M}\left[-\frac{1}{2}\mathrm{tr}\left[\alpha^{i}\sum_{j=1}^{M}{\left(\alpha^{j,i}\right)}^{-2}S_{{\vec{f}}_{t}^{j,i}}\right]+\log N\left({\vec{x}}_{t}^{i}|\alpha^{i}\sum_{j=1}^{M}{\left(\alpha^{j,i}\right)}^{-1}{\vec{\mu }}_{{\vec{f}}_{t}^{j,i}},I\alpha^{i}\right)\right] \end{array}$$

(A24) $$\begin{array}{cc} {\vec{\mu }}_{{\vec{f}}_{t}^{j,i}} & =K_{{\vec{x}}_{-t}^{j},{\vec{z}}_{:}^{j,i}}^{j,i}{\left(K_{{\vec{z}}_{:}^{j,i},{\vec{z}}_{:}^{j,i}}^{j,i}\right)}^{-1}{\vec{u}}_{:}^{j,i} \end{array}$$

(A25) $$\begin{array}{cc} S_{{\vec{f}}_{t}^{j,i}} & =K_{{\vec{x}}_{-t}^{j},{\vec{x}}_{-t}^{j}}^{j,i}-K_{{\vec{x}}_{-t}^{j},{\vec{z}}_{:}^{j,i}}^{j,i}{\left(K_{{\vec{z}}_{:}^{j,i},{\vec{z}}_{:}^{j,i}}^{j,i}\right)}^{-1}K_{{\vec{x}}_{-t}^{j},{\vec{z}}_{:}^{j,i}}^{j,i} \end{array}$$

(A26) $$\begin{array}{cc} \alpha^{i} & ={\left(\sum_{j=1}^{M}{\left(\alpha^{j,i}\right)}^{-1}\right)}^{-1} \end{array}$$

where K are kernel matrices, obtained by evaluating the kernel function at the pairs of points indicated by the subscripts. Equations (A24) and (A25) follow from the standard formulas for conditional Gaussians (see, e.g., [45], Identities 352–254). Equation (A26) follows from the product-of-experts construction (see Section 3, Equation (1)).

The next step in the evaluation of Equation (A22) is integrating A over $d{\vec{x}}_{t}^{:}$ and ${\vec{x}}_{-t}^{:}$, which requires averaging kernel matrices. The results of this averaging are denoted by Ψ (see Appendix B and [16] for a derivation). We denote $\Psi_{0}^{j,i}\left({\vec{x}}_{-t}^{j}\right)=\int d{\vec{x}}_{-t}^{j}q\left({\vec{x}}_{-t}^{j}\right)K_{{\vec{x}}_{-t}^{j},{\vec{x}}_{-t}^{j}}^{j,i}$, etc.:

(A27) $$\begin{array}{cc} B & =\int q\left({\vec{x}}_{t}^{:}\right)q\left({\vec{x}}_{-t}^{:}\right)\;A\;d{\vec{x}}_{t}^{:}\;d{\vec{x}}_{-t}^{:}\\ & =\sum_{i=1}^{M}\left(-\frac{1}{2}\alpha_{i}\sum_{j=1}^{M}{\left(\alpha^{j,i}\right)}^{-2}\mathrm{tr}\left[\Psi_{0}^{j,i}\left({\vec{x}}_{-t}^{j}\right)-{\left(K_{{\vec{z}}_{:}^{j,i},{\vec{z}}_{:}^{j,i}}^{j,i}\right)}^{-1}\Psi_{2}^{j,i}\left({\vec{x}}_{-t}^{j}\right)\right]\right.\\ & -Q_{i}\log\sqrt{2\pi \alpha^{i}}-\frac{1}{2}{\left(\alpha^{i}\right)}^{-1}\left[\mathrm{tr}\left(S_{{\vec{x}}_{t}^{i}}\right)+{\vec{\mu }}_{{\vec{x}}_{t}^{i}}^{T}{\vec{\mu }}_{{\vec{x}}_{t}^{i}}\right]\\ & +{\vec{\mu }}_{{\vec{x}}_{t}^{i}}^{T}\left(\sum_{j=1}^{M}{\left(\alpha^{j,i}\right)}^{-1}\Psi_{1}^{j,i}\left({\vec{x}}_{-t}^{j}\right){\left(K_{{\vec{z}}_{:}^{j,i},{\vec{z}}_{:}^{j,i}}^{j,i}\right)}^{-1}{\vec{u}}_{:}^{j,i}\right)\\ & -\left.\frac{1}{2}\alpha^{i}\sum_{j=1}^{M}\sum_{k=1}^{M}{\left(\alpha^{j,i}\right)}^{-1}{\left(\alpha^{k,i}\right)}^{-1}{\vec{u}}_{:}^{j,i}{}^{T}{\left(K_{{\vec{z}}_{:}^{j,i},{\vec{z}}_{:}^{j,i}}^{j,i}\right)}^{-1}\Psi_{2}^{j,k,i}\left({\vec{x}}_{-t}^{j},{\vec{x}}_{-t}^{k}\right){\left(K_{{\vec{z}}_{:}^{k,i},{\vec{z}}_{:}^{k,i}}^{k,i}\right)}^{-1}{\vec{u}}_{:}^{k,i}\right) \end{array}$$

Next, we sum this expression over time points:

(A28) $$\begin{array}{cc} C & =\sum_{t=1}^{T}B\\ & =\sum_{i=1}^{M}\left(\sum_{t=1}^{T}\left[-\frac{1}{2}\alpha^{i}\sum_{j=1}^{M}{\left(\alpha^{j,i}\right)}^{-2}\mathrm{tr}\left[\Psi_{0}^{j,i}\left({\vec{x}}_{-t}^{j}\right)-{\left(K_{{\vec{z}}_{:}^{j,i},{\vec{z}}_{:}^{j,i}}^{j,i}\right)}^{-1}\Psi_{2}^{j,i}\left({\vec{x}}_{-t}^{j}\right)\right]\right.\right.\\ & -\left.\log\sqrt{2\pi \alpha^{i}}-\frac{1}{2}{\left(\alpha^{i}\right)}^{-1}\left[\mathrm{tr}\left(S_{{\vec{x}}_{t}^{i}}\right)+{\vec{\mu }}_{{\vec{x}}_{t}^{i}}^{T}{\vec{\mu }}_{{\vec{x}}_{t}^{i}}\right]\right]\\ & +\sum_{j=1}^{M}{\left(\alpha^{j,i}\right)}^{-1}\left[\sum_{t=1}^{T}{\vec{\mu }}_{{\vec{x}}_{t}^{i}}^{T}\Psi_{1}^{j,i}\left({\vec{x}}_{-t}^{j}\right)\right]{\left(K_{{\vec{z}}_{:}^{j,i},{\vec{z}}_{:}^{j,i}}^{j,i}\right)}^{-1}{\vec{u}}_{:}^{j,i}\\ & -\left.\frac{1}{2}\alpha^{i}\sum_{j=1}^{M}\sum_{k=1}^{M}{\left(\alpha^{j,i}\right)}^{-1}{\left(\alpha^{k,i}\right)}^{-1}{\vec{u}}_{:}^{j,i}{}^{T}{\left(K_{{\vec{z}}_{:}^{j,i},{\vec{z}}_{:}^{j,i}}^{j,i}\right)}^{-1}\left[\sum_{t=1}^{T}\Psi_{2}^{j,k,i}\left({\vec{x}}_{-t}^{j},{\vec{x}}_{-t}^{k}\right)\right]{\left(K_{{\vec{z}}_{:}^{k,i},{\vec{z}}_{:}^{k,i}}^{j,i}\right)}^{-1}{\vec{u}}_{:}^{k,i}\right) \end{array}$$

For every part i∈1…M, we stack up the ${\vec{u}}_{:}^{:,i}$ into ${\vec{u}}^{i}$ (first by IV-index k=1,…,K, and then by part index such that ${\vec{u}}_{k}^{j,i}={\left({\vec{u}}^{i}\right)}_{Q^{i}\cdot \left(K\cdot j+k\right)+q^{i}+1}$) and construct a large block matrices $F^{i}$ and stacked vector $G^{i}$ with block elements

(A29) $$\begin{array}{cc} F_{j,k}^{i} & =\alpha^{i}\alpha_{j,i}^{-1}\alpha_{k,i}^{-1}{\left(K_{{\vec{z}}_{:}^{j,i},{\vec{z}}_{:}^{j,i}}^{j,i}\right)}^{-1}\left[\sum_{t=1}^{T}\Psi_{2}^{j,k,i}\left({\vec{x}}_{-t}^{j},{\vec{x}}_{-t}^{k}\right)\right]{\left(K_{{\vec{z}}_{:}^{k,i},{\vec{z}}_{:}^{k,i}}^{k,i}\right)}^{-1} \end{array}$$

(A30) $$\begin{array}{cc} G_{j}^{i} & ={\left(\alpha_{j,i}^{-1}\left[\sum_{t=1}^{T}{\vec{\mu }}_{{\vec{x}}_{t}^{i}}^{T}\Psi_{1}^{j,i}\left({\vec{x}}_{-t}^{j}\right)\right]{\left(K_{{\vec{z}}_{:}^{j,i},{\vec{z}}_{:}^{j,i}}^{j,i}\right)}^{-1}\right)}^{T} \end{array}$$

For j≠k: $\Psi_{2}^{j,k,i}\left({\vec{x}}_{-t}^{j},{\vec{x}}_{-t}^{k}\right)=\Psi_{1}^{j,i}\left({\vec{x}}_{-t}^{j}\right)\Psi_{1}^{k,i}\left({\vec{x}}_{-t}^{k}\right)$. Otherwise, $\Psi_{2}^{j,j,i}\left({\vec{x}}_{-t}^{j},{\vec{x}}_{-t}^{j}\right)=\Psi_{2}^{j,i}\left({\vec{x}}_{-t}^{j}\right)$ . We rewrite C as a quadratic form in the stacked augmenting IVs ${\vec{u}}^{i}$ to facilitate closed-form optimization of the dynamics ELB in Equation (A22) with respect to the stacked IV density $q({\vec{u}}^{:})$ using variational calculus, as described in Appendix A:

(A31) $$\begin{array}{cc} C & =\sum_{i=1}^{M}\left[-\frac{1}{2}{\vec{u}}^{iT}F^{i}{\vec{u}}^{i}+{\vec{u}}^{iT}G^{i}+H^{i}\right]\\ & =\sum_{i=1}^{M}\left[-\frac{1}{2}{\left({\vec{u}}^{i}-F^{i-1}G^{i}\right)}^{T}F^{i}\left({\vec{u}}^{i}-F^{i-1}G^{i}\right)+\frac{1}{2}G^{iT}F^{i-1}G^{i}+H^{i}\right] \end{array}$$

(A32) $$\begin{array}{cc} C & =\sum_{i=1}^{M}C^{i} \end{array}$$

(A33) $$\begin{array}{cc} C^{i} & =-\frac{1}{2}{\left({\vec{u}}^{i}-F^{i-1}G^{i}\right)}^{T}F^{i}\left({\vec{u}}^{i}-F^{i-1}G^{i}\right)+\frac{1}{2}G^{iT}F^{i-1}G^{i}+H^{i} \end{array}$$

(A34) $$\begin{array}{cc} H^{i} & =\sum_{t=1}^{T}\left[-\frac{1}{2}\alpha_{i}\sum_{j=1}^{M}{\left(\alpha_{j,i}^{-1}\right)}^{2}\mathrm{tr}\left[\Psi_{0}^{j,i}\left({\vec{x}}_{-t}^{j}\right)-{\left(K_{{\vec{z}}_{:}^{j,i},{\vec{z}}_{:}^{j,i}}^{j,i}\right)}^{-1}\Psi_{2}^{j,i}\left({\vec{x}}_{-t}^{j}\right)\right]\right.\\ & -\left.\log\sqrt{2\pi \alpha^{i}}-\frac{1}{2}\alpha_{i}^{-1}\left[\mathrm{tr}\left(S_{{\vec{x}}_{t}^{i}}\right)+{\vec{\mu }}_{{\vec{x}}_{t}^{i}}^{T}{\vec{\mu }}_{{\vec{x}}_{t}^{i}}\right]\right] \end{array}$$

After this optimization, we can write the dynamics ELBO using $p\left({\vec{u}}^{i}\right)=\prod_{j=1}^{M}p\left({\vec{u}}_{:}^{j,i}\right)=\prod_{j=1}^{M}N\left({\vec{u}}^{j,i}|0,K_{{\vec{z}}_{:}^{j,i},{\vec{z}}_{:}^{j,i}}\right)=N\left({\vec{u}}^{i}|0,K_{{\vec{z}}_{:}^{:,i},{\vec{z}}_{:}^{:,i}}\right)$ where $K_{{\vec{z}}_{:}^{:,i},{\vec{z}}_{:}^{:,i}}$ is a block-diagonal covariance matrix with the blocks given by the individual $K_{{\vec{z}}_{:}^{j,i},{\vec{z}}_{:}^{j,i}}$:

(A35) $$\begin{array}{cc} L_{dyn}\left(\Theta \right) & \geq \log\int p\left({\vec{u}}_{:}^{:,:}\right)\exp\left(C\right)d{\vec{u}}_{:}^{:,:}+H\left(q\left({\vec{x}}_{:}^{:}\right)\right)\\ & =\log\prod_{i=1}^{M}\int p\left({\vec{u}}^{i}\right)\exp\left(C^{i}\right)d{\vec{u}}^{i}+H\left(q\left({\vec{x}}_{:}^{:}\right)\right)\\ & =\sum_{i=1}^{M}\left[\log\int p\left({\vec{u}}^{i}\right)\exp\left(-\frac{1}{2}{\left({\vec{u}}^{i}-F^{i-1}G^{i}\right)}^{T}F^{i}\left({\vec{u}}^{i}-F^{i-1}G^{i}\right)\right)d{\vec{u}}^{i}+\frac{1}{2}G^{iT}F^{i-1}G^{i}+H^{i}\right]+H\left(q\left({\vec{x}}_{:}^{:}\right)\right)\\ & =\sum_{i=1}^{M}\left[-\log\sqrt{{\left(2\pi \right)}^{\dim(F^{i-1})}\left|F^{i-1}+K_{{\vec{z}}_{:}^{:,i},{\vec{z}}_{:}^{:,i}}\right|}-\frac{1}{2}G^{iT}F^{i-1}{\left(F^{i-1}+K_{{\vec{z}}_{:}^{:,i},{\vec{z}}_{:}^{:,i}}\right)}^{-1}F^{i-1}G^{i}+\log Z\left(F^{i-1}\right)\right]\\ & +\sum_{i=1}^{M}\left[\frac{1}{2}G^{iT}F^{i-1}G^{i}+H^{i}\right]+H\left(q\left({\vec{x}}_{:}^{:}\right)\right) \end{array}$$

This is the expression which we optimize with respect to $q({\vec{x}}_{:}^{:})$ and $q({\vec{u}}_{:}^{:,:})$. Since the stacked dynamics IVs ${\vec{u}}^{i}$ do not interact across parts in this expression (Line 2), it follows that density $q({\vec{u}}_{:}^{:,:})$ factorizes across parts. Their optimal density for each part is given by (cf. Equation (A6), *Z* is the normalization constant of the multivariate Gaussian):

(A36) $$\begin{array}{cc} q({\vec{u}}^{i}) & =\frac{1}{Z}p\left({\vec{u}}^{i}\right)\exp\left(C^{i}\right)\\ & =\frac{1}{Z}N\left({\vec{u}}^{i}|0,K_{{\vec{z}}_{:}^{:,i},{\vec{z}}_{:}^{:,i}}\right)\exp\left(-\frac{1}{2}{\left({\vec{u}}^{i}-F^{i-1}G^{i}\right)}^{T}F^{i}\left({\vec{u}}^{i}-F^{i-1}G^{i}\right)\right)\\ & =N({\vec{u}}^{i}|{\left(K_{{\vec{z}}_{:}^{:,i},{\vec{z}}_{:}^{:,i}}^{-1}+F^{i}\right)}^{-1}G^{i},{\left(K_{{\vec{z}}_{:}^{:,i},{\vec{z}}_{:}^{:,i}}^{-1}+F^{i}\right)}^{-1}) \end{array}$$
